# Combinational Growth Factor and Gas Delivery for Thrombosis Prevention

**DOI:** 10.3390/biom12111715

**Published:** 2022-11-19

**Authors:** Huan Cao, Xuejuan Xu, Fuyu Zhu, Yanhui Sheng

**Affiliations:** 1 Department of Cardiology, the Affiliated Suzhou Hospital of Nanjing Medical University, Suzhou Municipal Hospital, Gusu School, Nanjing Medical University, Suzhou 215008, China; 2 Department of Cardiology, Changshu Hospital, Nanjing University of Chinese Medicine, Changshu 215500, China; 3 Department of Cardiology, the First Affiliated Hospital of Nanjing Medical University, Nanjing 210029, China

**Keywords:** dual delivery, cell recruitment, re-endothelialization, NO generation

## Abstract

Cardiovascular stents enable the rapid re-endothelialization of endothelial cells (ECs), and the constant suppression of smooth muscle cell (SMC) proliferation has been proved to effectively prevent thrombosis. However, the development and application of such stents are still insufficient due the delayed re-endothelialization progress, as well as the poor durability of the SMC inhibition. In this paper, we developed a mussel-inspired coating with the ability for the dual delivery of both growth factor (e.g., platelet-derived growth factor, PDGF) and therapeutic gas (e.g., nitric oxide, NO) for thrombosis prevention. We firstly synthesized the mussel-inspired co-polymer (DMHM) of dopamine methacrylamide (DMA) and hydroxyethyl methacrylate (HEMA) and then coated the DMHM on 316L SS stents combined with Cu^II^. Afterwards, we immobilized the PDGF on the DMHM-coated stent and found that the PDGF could be released in the first 3 days to enhance the recruitment, proliferation, and migration of human umbilical vein endothelial cells (HUVECs) to promote re-endothelialization. The Cu^II^ could be “sealed” in the DMHM coating, with extended durability (2 months), with the capacity for catalyzed NO generation for up to 2 months to suppress the proliferation of SMCs. Such a stent surface modification strategy could enhance the development of the cardiovascular stents for thrombosis prevention.

## 1. Introduction

Cardiovascular diseases (CVDs) are the main cause of chronic disability and premature death globally [1]. Clinically, the most commonly used therapy for CVDs is the cardiovascular stents intervention, including bare metal stents (BMSs) and drug-eluting stents (DESs) [2,3]. Different from the bare BMSs, the DESs can significantly lower the restenosis rate due to the localized drug delivery of biological agents to the targeted sites, with therapeutically effective drug concentration and the minimal systemic toxicity [4,5]. For example, the paclitaxel-loaded DESs could limit smooth muscle cell (SMC) proliferation for reduced late-stage in-stent restenosis (ISR) [6]. In addition, the sirolimus-loaded DESs could also arrest the smooth muscle cells by inhibiting the cell cycle progression [7]. This is because long-term excessive SMC proliferation could result in maladaptive neointimal proliferation, leading to restenosis. Unfortunately, these generally used drugs could uniformly suppress endothelial cell (EC) growth, with hampered re-endothelialization leading to the compromised efficacy of treating ISR [8,9]. To this end, the DESs could not completely eliminate the ISR, which occurs at a rate of between 5% and 10% after DESs implantation [10,11,12]. Moreover, the inevitable pathological response of stent implantation, such as EC layer damage, could cause leukocyte aggregation and inflammation, ultimately leading to thrombosis. It has been reported that rapid re-endothelialization following stent implementation (7–10 days), with accelerated EC migration and proliferation, is critical for thrombosis prevention [13]. The regenerated EC could serve as a barrier against platelet/leukocyte adhesion to inhibit ISR. Therefore, the optimized stent coating should allow for rapid re-endothelialization and then offer long-term SMC proliferation suppression to prevent thrombosis.

To date, to boost the safety and effectiveness of cardiovascular stents, significant efforts have been made to design and engineer the stent surface. For example, the mussel-inspired polydopamine (PDA) coatings present universal adhesiveness to various surfaces in physiological underwater conditions [14]. The existence of multiple functional groups could also enable the further modification of the surfaces [15]. In addition, such PDA coatings present superior biocompatibility, with promoted EC adhesion, proliferation, and migration [16]. However, the homogeneity and robustness of the PDA coating may significantly affect the performance of the cell behaviors. Growth factors, such as platelet-derived growth factor (PDGF), could improve EC recruitment, adhesion, migration, and proliferation, while reducing the severity of the initial local inflammatory response after stent implantation [17]. Nevertheless, the effective immobilization and controllable release of PDGF is still the main problem regarding stent coating preparation. In recent years, the nitric oxide (NO)-releasing coatings have attracted substantial attention. The NO can not only suppress the SMC proliferation, but it can also promote EC migration and growth [8]. For instance, researchers have demonstrated that the Cu^II^ exhibits a biomimicking glutathione peroxidase (GPx) property which could catalyze NO production by degrading endogenous NO donors in the bloodstream. Thus, the Cu^II^ could serve as the NO generation catalyzer in the NO-releasing coatings [14]. However, these NO-producing coatings are not adequate to induce early re-endothelialization quickly. More importantly, they are unable to realize the durable NO release for effective long-term SMCs inhibition. Thus, the development of novel stent coatings with rapid early re-endothelialization and long-term NO release for SMC inhibition is highly demanded in clinic.

**Figure 1:**
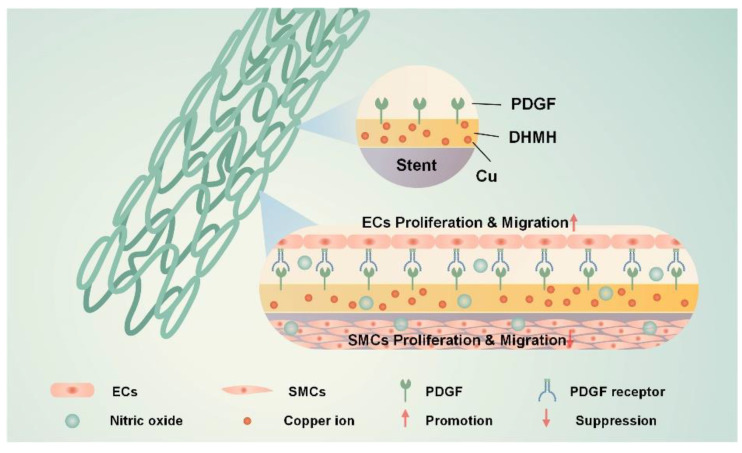
The schematic showing the surface modification strategy of the cardiovascular stent with the dual delivery of PDGF and NO.

In this paper, we developed a mussel-inspired coating with the capability of the dual delivery of both PDGF and NO for thrombosis prevention (Figure 1). We firstly synthesized the mussel-inspired co-polymer (DMHM) of dopamine methacrylamide (DMA) and hydroxyethyl methacrylate (HEMA), based on methods provided in the literature [18]. Then, we dip coated the 316L SS stents with the DMHM and Cu^II^ combined to embed the Cu^II^ into the DMHM layer. This is because the DMHM could be strongly coated on the 316L SS stents because of the presence of the adhesive catechol groups [19]. Interestingly, we found the Cu^II^ could be “sealed” in the DMHM coating during the DMHM coating assembling process, with long-term durability (2 months), which could realize the long-term NO regeneration. Then, we immobilized the PDGF on the DMHM-coated stent, with even distribution. We demonstrated that the PDGF could be released in the first 3 days to recruit the human umbilical vein endothelial cells (HUVECs) and enhance the HUVECs proliferation and migration, as well as promote endothelialization by enhancing the vascular endothelial growth factor (VEGF) production to induce the promoted ECs lineage commitment. In addition, due to the effective sealing of the CuII in the DMHM coating, it could catalyze NO production by degrading endogenous NO donors and realize long-term catalyzed NO generation for 2 months. The generated NO could suppress the SMCs proliferation by activating the cGMP pathway [8]. Therefore, such a smart combinational release of PDGF and NO could achieve rapid re-endothelialization and long-term ISR inhibition [8,20]. We hope that such a stent surface modification strategy will enhance the development of cardiovascular stents for thrombosis prevention.

## 2. Materials and Methods

### 2.1. Synthesis of the DMHM

The DMHM was synthesized based on the methods in a previous study [18]. Briefly, 3.4 mL hydroxyethyl methacrylate (HEMA, Macklin reagent, China), 1.2 g dopamine methacrylamide (DMA, Macklin reagent, China), and 84 mg azobisisobutyronitrile (AIBN, Macklin reagent, China) were added to the flask with 20 mL dimethylformamide (DMF, Macklin reagent, China). The mixture was co-polymerized at 60 °C, with nitrogen protection. The resultant product was precipitated by diethyl ether and dried in an oven. Fourier transform infrared (FTIR) spectroscopy was used for the copolymer evaluation [8].

### 2.2. Preparation and Characterization of the Coatings

For the preparation of the DMHM-Cu^II^ (D-Cu) coatings, the DMHM was first dissolved in the ethanol with a 4 mg/mL concentration. Then, 10 µg/mL CuCl_2_ (Macklin reagent, China) was added to the DMHM solution. The mirror-polished 316L SS foils (Wenhou Metal Co., Ltd., Hefei, China) were cleaned by acetone, ethanol, and DI water, followed by thoroughly drying them in air. Then, the D-Cu coated samples were immersed in the phosphate-buffered saline (PBS, pH 7.4, Thermo Fisher, Suzhou, China) with 1 µg/mL PDGF for 12 h at 37 °C to obtain the final PDGF immobilized samples (D-Cu-P). Energy-dispersive X-ray spectroscopy (EDX, Hitachi, Tokyo, Japan) was used to image and quantify the Cu element distribution and content [21,22]. The optical contact angle measuring and contour analysis systems (DataPhysics Instruments, Filderstadt, Germany) were used to evaluate the water contact angle (WCA) of the samples with 10 µL deionized (DI) water [21]. The release kinetics of the PDGF were analyzed using an enzyme-linked immunosorbent assay (ELISA) kit for PDGF, following the manufactory’s protocol [23].

### 2.3. Biocompatibility and Cell Recruitment Evaluations

The HUVECs (Cyagen, Suzhou, China) was used in these experiments to test the viability, adhesion, and proliferation of HUVECs on the prepared samples. Briefly, HUVECs (5 × 10^4^ cells/cm^2^) were seeded onto different samples and cultured in endothelial cell medium (ECM, Sciencell, Carlsbad, CA, USA) supplemented with fetal bovine serum (10%, Gibco, Suzhou, China) and Penicillin-Streptomycin (1%, Gibco, Suzhou, China). After 1, 2, and 3 days of incubation, the Live/Dead kit (Thermo Fisher, Suzhou, China) and a CCK-8 quantification assay (Beyotime, Shanghai, China) were used to study the adhesion, viability, and proliferation of HUVECs [19].

### 2.4. Re-Endothelialization Evaluations

To evaluate the re-endothelialization efficacy of the coating, the cell migration of HUVECs was analyzed with HUVECs (5 × 10^4^ cells/cm^2^). After the cell confluence, a p200 pipette tip was adopted to scratch the cell monolayer. After 24 h of incubation, the HUVECs were stained with crystal violet for observation. The wound healing rate was calculated by: H = (H0 − H1)/H0 × 100%, where H0 and H1 were the initial and final scratch areas, respectively [24]. Then, a tube formation assay was further adopted. In detail, round samples (10 mm in diameter) were placed in the bottom of the 24-well plate and pre-coated with Matrigel. The HUVECs (5 × 10^4^ cells/cm^2^) were deposited onto the Matrigel-coated samples and incubated at 37 °C in 5% CO_2_. After predetermined time points, we observed the tube formation with Calcein AM staining (Beyotime, Shanghai, China). We also quantified the total branch length and the average number of junctions, based on methods described in the literature [25].

### 2.5. Catalyzed NO Generation and SMCs Inhibition Evaluations

We adopted human umbilical artery smooth muscle cells (HUASMCs) for the evaluation of the SMCs inhibition efficacy of coating, cell adhesion, and proliferation. HUASMCs (2 × 10^4^ cells/cm^2^) were seeded onto different samples and cultured in DMEM/F12 medium (Thermo Fisher, Suzhou, China) supplemented with fetal bovine serum (10%, Gibco, China) and Penicillin-Streptomycin (1%, Gibco, China). After culturing for 2 h, 24 h, and 72 h, the SMCs were stained with Calcein AM (Beyotime, Shanghai, China) and observed by fluorescence microscopy (Nikon, Tokyo, Japan). The Cu^II^ ions in the D-Cu and D-Cu-P coatings (sealed by the DMHM co-polymer) can slowly release and catalyze the NO generation, with help of an NO donor (e.g., RSNO) and glutathione (GSH). Briefly, the D-Cu and D-Cu-P coated samples were immersed in 5 mL of PBS supplemented with 10 μM RSNO and 10 μM GSH. The NO generation occurred due to the degradation of added NO donors catalyzed by the Cu^II^ ions. The NO generation was quantified by the Griess method, in which the nitrite concentration was tested at predetermined time points [26].

### 2.6. Statistical Analysis

All the data were demonstrated as mean ± standard deviation (SD), with n = 3, unless otherwise stated. Then, one-way and two-way analysis of variance and Tukey’s post hoc test were adopted to analysis the statistical significance (SPSS Statistics, IBM, Armonk, NY USA). A *p*-value < 0.05 was considered statistically significant.

## 3. Results and Discussions

### 3.1. Preparation and Characterization of Coatings

**Figure 2:**
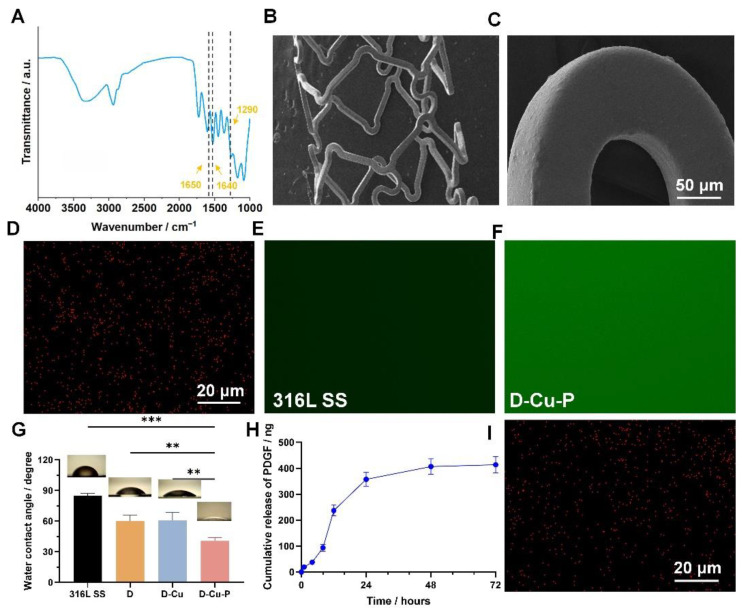
The evaluation of the surface modification of the 316L SS foils. (**A**) FTIR spectra of the synthesized DMHM (the dotted line indicated the position of specific peaks). (**B**,**C**) SEM evaluation of the stents after DMHM and Cu^II^ coating. (**D**) The EDX evaluation of the Cu element on the DMHM- and Cu^II^-coated samples. (**E**,**F**) The evaluation of the PDGF immobilization through the fluorescence intensity of FITC-BSA. (**G**) The contact angle evaluation of the prepared coatings. (**H**) The cumulative PDGF release of the PDGF immobilization samples. (**I**) The EDX evaluation of the Cu element after 2 months. ** *p* < 0.01 and *** *p* < 0.001 denote the statistical significance.

We firstly synthesized the DMHM polymer, as described in the literature, and used the FTIR to evaluate the synthesized DMHM (Figure 2A). We found that all the characteristic peaks of the DMHM could be observed at 1290 cm^−1^ (catechol groups), 1400–1600 cm^−1^ (benzene skeleton), and 1650 cm^−1^ (amide in DMHM), indicating the successful materials synthesis [27]. Afterwards, we prepared the DMHM-coated 316L SS foils, along with the Cu^II^ (D-Cu), through a one-pot coating process. We used scanning electron microscopy (SEM) to observe the coating, and we found the DMHM could form a homogenous coating on the stent (Figure 2B,C). We found that the Cu^II^ could be evenly distributed and sealed in the DMHM coating, as demonstrated by the energy-dispersive X-ray spectroscopy (EDX) evaluation (Figure 2D). Next, we further immobilized the PDGF onto the prepared D-Cu samples (D-Cu-P). We used the fluorescein isothiocyanate-labelled bovine serum albumin (FITC-BSA) as a model protein and found that the PDGF could be homogeneously distributed onto the D-Cu-P due to the possible Schiff-base reaction and the electrostatic interaction (Figure 2E,F) [28]. It has been widely reported that wettability plays a critical role in the cell adhesion. Thus, we evaluated the wettability of the prepared surface. We found that the DMHM-coated and D-Cu coatings are more hydrophilic compared to the naked samples. The immobilization of PDGF led to a further decrease in the contact angle, indicating higher hydrophilicity properties (Figure 2G). Such results are consistent with those in the literature, and could result from the hydrophilicity moieties on the DMHM and PDGF [29]. Then, we tested the release of Cu^II^ and PDGF from the prepared coatings. The release of PDGF was tested by an enzyme-linked immunosorbent assay (ELISA) kit for PDGF. After the PDGF immobilization, the PDGF could be released from the D-Cu-P coating for 3 days, without burst release, indicating the DMHM could realize the stable and controllable early release of the PDGF, which is of great importance for the early re-endothelialization process (Figure 2H) [23]. Moreover, we also tested the durability of Cu^II^ sealed in the DMHM coating, and we found that the Cu^II^ presented a similar distribution under EDX analysis, after 2 months (Figure 2I). The quantification of the Cu^II^ further revealed a decrease of around 1.71% of the Cu element after 2 months, indicating the existence of residual Cu^II^ (Figure 2I and Table 1). However, we also found that the Cu^II^ element concentration in coatings decreased around 78% after 2 months (from 2.19% to 0.48%). This could be attributed to the degradation of the DMHM coating, creating a durability concern regarding our coating after 2 months, which may require further evaluation.

### 3.2. Biocompatibility and Cell Recruitment Evaluations

**Figure 3:**
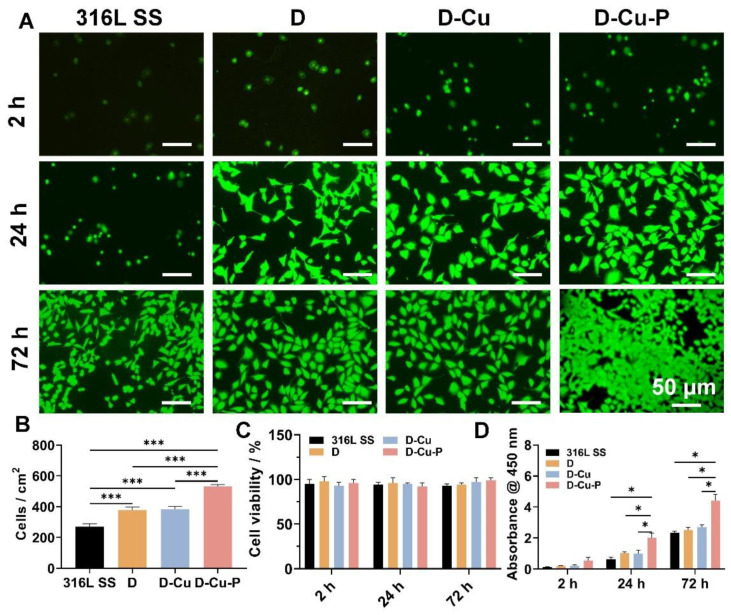
The biocompatibility and cell recruitment evaluations of the coated samples. (**A**) Live/dead staining of the HUVECs after 2, 24, and 72 h. (**B**) Cell adhesion rate, (**C**) viability, and (**D**) proliferation rate of the samples. * *p* < 0.05 and *** *p <* 0.001 denote the statistical significance.

We then performed the biocompatibility and cell recruitment evaluations. We used the human umbilical vein endothelial cells (HUVECs) as the model cells, since they are critical in the re-endothelialization process. After directly seeding the HUVECs onto different samples, all the DMHM-coated samples showed significantly higher cell recruitment rates after 2 h (Figure 3A,B). The D-Cu groups presented 1.4-fold initial cell numbers compared to the 316L SS groups. In addition, we also observed the cell viability and proliferation, and found that all the groups showed over 90% cell viability, and the Cu^II^ had no detrimental effect on the cell growth (Figure 3B). As expected, the D-Cu-P presented the highest cell proliferation rate among all the groups (1.6 and 1.9-fold proliferation compared to the 316L SS and D-Cu groups), indicating that the PDGF could enhance the proliferation of HUVECs (Figure 3C). These results indicated the excellent biocompatibility of our D-Cu-P coating, which would not have a harmful effect when the released Cu enters circulation. Such results are also consistent with those in the literature. In addition, our D-Cu-P coating could also effectively recruit HUVECs and promote cell growth [30,31].

### 3.3. Re-Endothelialization Capacity Evaluations

**Figure 4:**
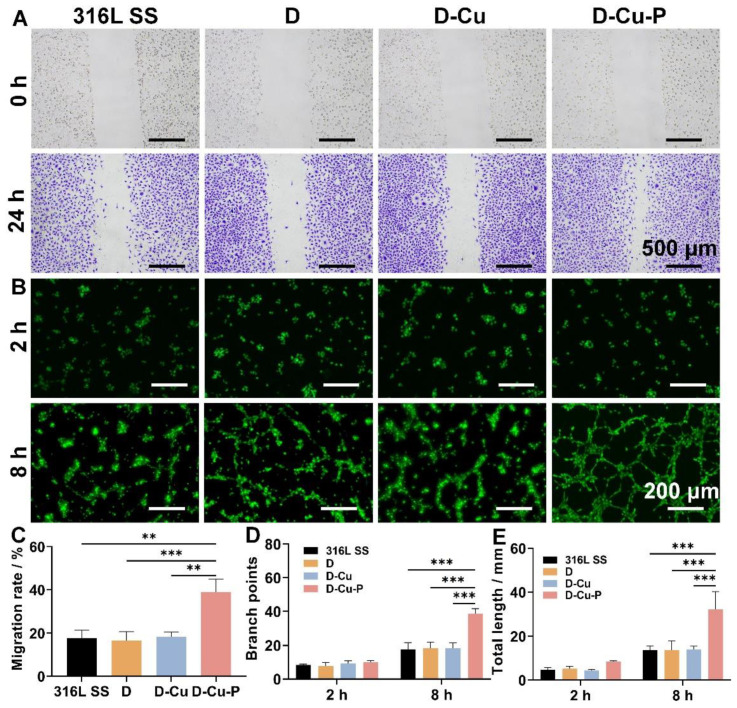
Re-endothelialization capacity evaluations of HUVECs. (**A**) HUVECs migration assay and (**B**) tube formation assay for the different coated samples. (**C**) The quantification of cell migration rate in the migration assay. The quantification of (**D**) branch points and (**E**) total length of tubulars in tube formation assay. ** *p < 0.01* and *** *p <* 0.001 denote the statistical significance.

Next, we moved on to the evaluation of the re-endothelialization capacity of our developed coatings. We firstly tested the HUVECs migration on our coatings because the enhanced cell migration is the key to accelerate re-endothelialization and prevent ISR. We found that the DMHM coating could enhance the HUVECs migration, which could be attributed to the increased cellular affinity after the DMHM modification. Notably, the D-Cu-P group, presented by the PDGF release, significantly increased the cell migration rate with a 2.2-fold increase in the wound healing rate compared to that of the 316L SS group (Figure 4A,C). Further, we also used the tube formation assay to validate the capacity of HUVECs to form capillary-like structures in a three-dimensional microenvironment. We found that the 316L SS group, the DMHM groups, and the D-Cu group showed a similar number of cells extending pseudopodia, with limited cell connections after 12 h (Figure 4B). However, the tubular structure was clearly visible in the D-Cu-P group. The quantification of the average tube length and branching points also revealed that the D-Cu-P group exhibited a 2.2- and 2.4-fold increase compared with the 316L SS group, revealing the superior tubulogenesis of HUVECs in the D-Cu-P group (Figure 4C,D). Since there is no NO donor in any of the experiments, the effect of Cu^II^ catalyzed NO generation could be ignored. To this end, these results collectively confirm that our D-Cu-P coating could realize re-endothelialization, along with the enhanced migration of the HUVECs, as well as tubulogenesis.

### 3.4. Catalyzed NO Generation and HUASMCs Inhibition Evaluations

**Figure 5:**
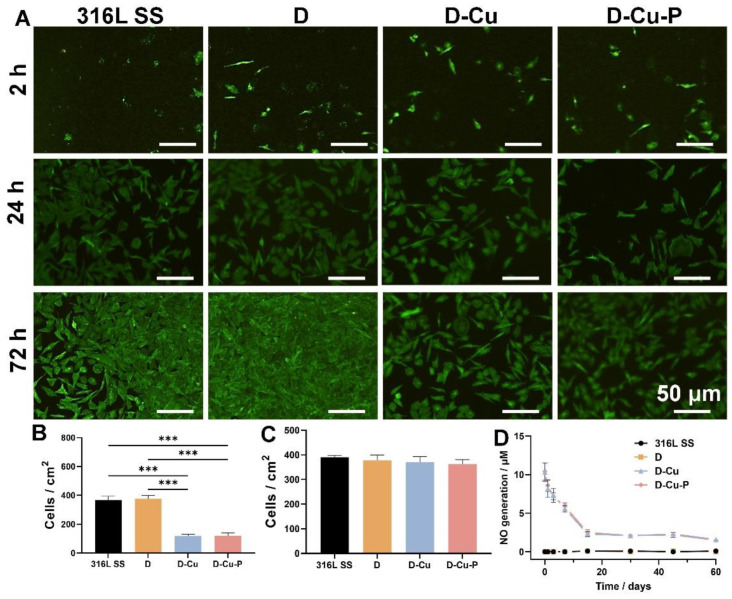
Catalyzed NO generation and SMCs inhibition evaluations. (**A**) Live/dead staining of the SMCs after 2, 24, and 72 h. (**B**) Cell adhesion rate, (**C**) viability, and (**D**) proliferation rate of the samples. *** *p* < 0.001 denote the statistical significance.

After demonstrating the rapid re-endothelialization performance, we then studied the catalyzed NO generation and SMCs inhibition, with or without the addition of NO donors (S-nitrosothiols (RSNO) and glutathione (GSH)) [32]. We used the HUASMCs as the model cells. We found that the D-Cu and D-Cu-P groups demonstrated a substantial inhibition of the HUASMCs compared with the naked 316L SS samples after 24 h with NO donor addition (Figure 5A). The quantification evaluation of the cell number further confirmed such an inhibition effect (Figure 5B). Previously, it has been reported that the PDGF is a significant stimulant of human smooth muscle cell growth due to the activation of PDGF-Rα and enhancing of the phosphorylation of ERK1/2 and Akt [33]. However, in our study, we found that such stimulation was neutralized by the robust NO generation catalyzed by Cu^II^. Such contradictory result could be attributed to the inhibition effects of the released NO, which could inhibit SMCs proliferation by cGMP-independent mechanisms [34]. However, such an inhibitory effect disappeared without the addition of the NO donor, indicating the potential role of NO generation in the fate modulation of the SMCs (Figure 5C). Thus, we then tested the NO regeneration of coatings with the NO donor. We found that the Cu^II^-bearing groups could generate NO, even after 2 months. In all, the exhibited long-term NO catalyzed formation is high favorable for the long-term anti-thrombotic effects (Figure 5D). Additionally, our Cu^II^-bearing coating, in place over 60 days, still catalyzed NO generation which is a longer period than that reported in previous studies also adopting Cu^II^ as a catalyzer. For example, the unbalanced magnetron sputtering of the Ti–Cu could only realize catalyzation up until 30 days of the Cu^II^ release [32]. Such an extended-release period of our coating demonstrated the excellent “sealing” effect of our proposed mussel-inspired co-polymer (DMHM), which could significantly enhance the durability up to 2 months and achieve the prolonged Cu^II^ catalyzing efficacy. Clinically, myocardial infarction is commonly associated with hyperglycemia, with increased glucose concentration. Thus, we noted the possibility that the Cu^II^ may possibly be reduced to Cu^I^ via such reducing agents (e.g., glucose) [35,36]. To this end, the long-term efficacy of Cu^II^ in our coating in real pathological condition requires further in-depth studies.

## 4. Conclusions

In this paper, we developed a mussel-inspired coating with dual delivery capabilities for PDGF and NO for use in thrombosis prevention. We synthesized the DMHM polymer followed by the coating on 316L SS stents in combination with Cu^II^. Afterwards, we immobilized the PDGF on the DMHM-coated stent, with even distribution. We found that the PDGF could be released in the first 7 days to recruit the HUVECs, and enhance HUVECs proliferation and migration to promote re-endothelialization. The Cu^II^ could be “sealed” in the DMHM coating, with the capacity for catalyzed NO generation for up to 2 months to suppress the proliferation of HUASMCs. We hope that such stent surface modification strategy will enhance the development of the cardiovascular stents for thrombosis prevention. However, there are some limitations of our present study. (1) The long-term efficacy of Cu^II^ in our coating in real pathological condition requires further in-depth studies. The Cu^II^ could be reduced by the reducing agents (e.g., glucose) present in a myocardial infarction condition, which may compromise its efficiency. (2) The Cu^II^ element concentration in the coatings decreased by around 78% after 2 months, and the durability of the coating after 2 months rquires further characterization. (3) The in vivo compatibility evaluation of the coating should be performed to further clarify the potential toxicity of the Cu^II^ element in our coating.

## Figures and Tables

**Table 1:** The relative chemical compositions of the DMHM-Cu^II^ coating initially and after 2 months.

Time (Days)	C (%)	N (%)	O (%)	Cu (%)
0	74.33 ± 2.34	11.29 ± 1.97	12.19 ± 1.33	2.19 ± 0.35
60	75.73 ± 3.14	11.47 ± 1.83	12.32 ± 1.02	0.48 ± 0.05

## Data Availability

The data are available upon reasonable request.
